# TLR1/2 Activation during Heterologous Prime-Boost Vaccination (DNA-MVA) Enhances CD8+ T Cell Responses Providing Protection against *Leishmania (Viannia)*

**DOI:** 10.1371/journal.pntd.0001204

**Published:** 2011-06-14

**Authors:** Asha Jayakumar, Tiago M. Castilho, Esther Park, Karen Goldsmith-Pestana, Jenefer M. Blackwell, Diane McMahon-Pratt

**Affiliations:** 1 Yale University School of Public Health, New Haven, Connecticut, United States of America; 2 Centre for Child Health Research, Telethon Institute for Child Health Research, University of Western Australia, Perth, Australia; University of Pennsylvania School of Medicine, United States of America

## Abstract

**Background:**

*Leishmania (Viannia)* parasites present particular challenges, as human and murine immune responses to infection are distinct from other *Leishmania* species, indicating a unique interaction with the host. Further, vaccination studies utilizing small animal models indicate that modalities and antigens that prevent infection by other *Leishmania* species are generally not protective.

**Methodology:**

Using a newly developed mouse model of chronic *L. (Viannia) panamensis* infection and the heterologous DNA prime – modified vaccinia virus Ankara (MVA) boost vaccination modality, we examined whether the conserved vaccine candidate antigen tryparedoxin peroxidase (TRYP) could provide protection against infection/disease.

**Results:**

Heterologous prime – boost (DNA/MVA) vaccination utilizing TRYP antigen can provide protection against disease caused by *L. (V.) panamensis*. However, protection is dependent on modulating the innate immune response using the TLR1/2 agonist Pam3CSK4 during DNA priming. Prime-boost vaccination using DNA alone fails to protect. Prior to infection protectively vaccinated mice exhibit augmented CD4 and CD8 IFNγ and memory responses as well as decreased IL-10 and IL-13 responses. IL-13 and IL-10 have been shown to be independently critical for disease in this model. CD8 T cells have an essential role in mediating host defense, as CD8 depletion reversed protection in the vaccinated mice; vaccinated mice depleted of CD4 T cells remained protected. Hence, vaccine-induced protection is dependent upon TLR1/2 activation instructing the generation of antigen specific CD8 cells and restricting IL-13 and IL-10 responses.

**Conclusions:**

Given the general effectiveness of prime-boost vaccination, the recalcitrance of *Leishmania (Viannia)* to vaccine approaches effective against other species of *Leishmania* is again evident. However, prime-boost vaccination modality can with modulation induce protective responses, indicating that the delivery system is critical. Moreover, these results suggest that CD8 T cells should be targeted for the development of a vaccine against infection caused by *Leishmania (Viannia)* parasites. Further, TLR1/2 modulation may be useful in vaccines where CD8 T cell responses are critical.

## Author Summary

*Leishmania (Viannia)* are the predominant agents of leishmaniasis in Latin America. Given the fact that leishmaniasis is a zoonosis, eradication is unlikely; a vaccine could provide effective prevention of disease. However, these parasites present a challenge and we do not fully understand what elements of the host immune defense prevent disease. We examined the ability of vaccination to protect against *L. (Viannia*) infection using the highly immunogenic heterologous prime-boost (DNA-modified vaccinia virus) modality and a single *Leishmania* antigen (TRYP). Although this mode of vaccination can induce protection against other leishmaniases (cutaneous, visceral), no protection was observed against *L. (V.) panamensis*. However, we found that if the vaccination was modified and the innate immune response was activated through Toll-like receptor1/2(TLR1/2) during the DNA priming, vaccinated mice were protected. Protection was dependent on CD8 T cells. Vaccinated mice had higher CD8 T cell responses and decreased levels of cytokines known to promote infection. Given the long-term persistence of CD8 T cell memory, these findings are encouraging for vaccine development. Further, these results suggest that modulation of TLR1/2 signaling could improve the efficacy of DNA-based vaccines, especially where CD8 T cell activation is critical, thereby contributing to effective and affordable anti parasitic vaccines.

## Introduction

Traditionally, vaccination against cutaneous leishmaniasis (CL) has involved leishmanization (inoculation of live *Leishmania*), which has been practiced throughout the Middle East and was employed in government sponsored vaccination programs both in Israel and Russia. However, safety and standardization issues discouraged further use of live vaccination [1], [2]. Subsequently, killed *Leishmania* promastigotes have been examined with some but limited efficacy in clinical trials [3], [4]. Consequently, leishmaniasis vaccine efforts have focused on the use of live attenuated vaccines [5], [6] and also defined molecular vaccines and delivery systems [7].

An optimal vaccine against cutaneous leishmaniasis would consistently provide protection against the various disease-causing species. However, studies indicate that distinct *Leishmania* species elicit different responses in their hosts, suggesting that a uniform approach might be challenging. Although a Th1-like response is considered to lead to disease resolution, the mechanisms contributing to protection across the species are not well characterized/understood. In particular, the *Leishmania (Viannia)* subgenus is phylogenetically divergent from the *Leishmania (Leishmania)* subgenus [8], [9], [10]. Members of the *L. (Viannia)* subgenus can generate a hyperinflammatory response that fails to resolve [11], [12], [13], . *L. (V).panamensis* elicits a mixed Th1/Th2 and non-resolving hyperinflammatory response to infection in humans [17], [18].

Consistent with this, vaccine studies attempting to demonstrate immunological protection against *L. (Viannia)* parasites [19], [20], [21] using a murine model, have met with limited success. Salay *et al*. [19] tested four different highly conserved leishmanial antigens (DNA or recombinant protein) along with adjuvants that have protected against infection with other species causing CL (*L. mexicana*, *L. amazonensis*, *L. major*). However, strong antigen-specific IFNγ production by immunized mice failed to translate into protection against *L. (Viannia) braziliensis* infection. As a result this study suggested investigation of alternate immunization strategies to protect against *L. (Viannia)* parasites. Similarly, antigens demonstrated to protect against visceral leishmaniasis [21] failed to protect against *L. (Viannia) braziliensis*. Recently, partial protection was [20] demonstrated against *L.(V.) braziliensis* by utilizing an attenuated centrin-deficient *L. donovani* strain. Taken as a whole these studies might suggest that defined antigens may not provide protection against *L. (Viannia)*. However, vaccine delivery systems are critical to determining the elicited immune response and therefore can determine protection provided for an antigen. In particular, as the mechanisms involved in disease resolution for *L. (Viannia)* are not well understood, further investigation of delivery systems/antigens is warranted and may ultimately provide insight into immune mechanisms leading to healing. Hence we explored other immunization methods to induce protection against *L. (V.) panamensis*, using a newly developed murine model for chronic disease [22].

Herein we report for the first time that heterologous prime (DNA) -boost (modified vaccinia virus Ankara  =  MVA) modality using the single antigen tryparedoxin peroxidase (TRYP) and including the TLR1/2 agonist N-palmitoyl-S-[2,3-bis(palmitoyloxy)-(2RS)-propyl]-[R]-cysteinyl-[S]-seryl-[S]-lysyl-[S]-lysyl-[S]-lysyl-[S]-lysine (Pam3CSK4) as adjuvant during DNA priming is effective in achieving protection against *L.(V.) panamensis.* Markedly, prime boost immunization in the absence of Pam3CSK4 did not elicit protection thereby implicating a strategic role for Pam3CSK4 in achieving protection. Pam3CSK4 appears to direct heightened CD4 and CD8 T memory cell responses and reduced levels of IL-10 and IL-13, which ultimately results in significant protection against *L.(V.) panamensis.* Furthermore CD8 cells, but interestingly not CD4 T cells, are crucial in mediating the protection induced, indicating that CD8 T cell responses may be critical for vaccine development against *L. (Viannia)* parasites.

## Materials and Methods

### *Leishmania* culture and infection

*L. (V.) panamensis* was grown and cultured into infective stage parasites as described previously [22]. Briefly *L. (V.) panamensis* was grown in Schneider's Medium supplemented with 20% heat inactivated FCS and 17.5 µg/mL gentamycin (GIBCO BRL). Promastigotes were grown at 22°C. Live late stationary phase (15–21 days in culture) promastigotes were harvested for infection using a step percoll gradient (Sigma Chemical Co.) in PBS containing 20 mM EDTA. Washed parasites (5×10^4^) were used to infect mice in the top of the right hind foot.

### Animals

Female BALB/c mice (5 to 6 weeks old) were purchased from the NCI. All mice were housed in Yale University School of Medicine facilities, which are American Association for Accreditation of Laboratory Animal Care (AAALAC) accredited and USDA registered animal facilities. The experiments were approved by Yale University Committee on Use and Care of Animals (Assurance number A3230-01).

### Plasmids, recombinant proteins, and vaccinia virus

TRYP and p36 (LACK) genes were cloned into pVAX (Invitrogen, CA) and pCI-neo (Promega, WI) vectors respectively. Plasmids were purified using Qiagen Endofree Plasmid Giga kit (Qiagen, CA). Empty plasmid was used for the controls. Plasmid preparations were tested for endotoxin by Limulus Amebocyte Lysate test (Lonza, MD); less than 0.1 ng LPS per 100 µg of plasmid was present in preparations employed for vaccination. The p36 and TRYP recombinant proteins were expressed using a histidine-tag construct that was cloned into pRSET A vector kindly provided by Dr. Larraga (Centro de Investigaciones Biológicas, Spain) and pET-15b vector, respectively. Recombinant protein was purified using PrepEase Histidine-Tagged Protein Purification kit (USB, OH) and endotoxin was removed as described [23]. Coomassie blue staining of SDS-PAGE analysis of recombinant antigen was used to determine protein purity. Vaccinia virus Ankara (MVA) expressing TRYP and LACK were prepared as previously described [24], [25].

### Vaccination and immunodepletion studies

For adjuvant evaluation, mice (3/group) received two intra dermal injections of p36 DNA (100 ug in 100 ul) per vaccination with or without adjuvants (α-GalCer (1 ug), LPS (10 ug), CpG (50 ug), Pam3CSK4 (10 ug), MALP-2 (0.5 ug)). After an interval of 3 weeks, mice were boosted using the same DNA-adjuvant combination. Splenocytes from the vaccinated mice were evaluated by *in vitro* cytokine production 4 weeks after the final immunization. This experiment was done twice and 3 mice per group were sufficient to achieve statistical significance and evaluation of data.

In the case of DNA-vaccinia virus (MVA) prime-boost vaccination, 2 weeks after the priming immunization with TRYP(100 ug/100 ul) ± Pam3CSK4, mice (8 to 10/group) were boosted intraperitoneally with 3×10^6^ PFU per mouse of MVA-TRYP. This was followed by infection with 5×10^4^ late stationary phase *L. (V.) panamensis* promastigotes 6 weeks after the MVA boost. Lesion development was monitored by measuring the thickness of the infected and uninfected feet using a dial gauge caliper (Starrett Thickness Gauge). Parasite burdens in the infected foot and draining lymph node (DLN) were determined by limiting dilution analysis as described previously [26], [27]. As parasite burden changes in the infected foot and associated DLN were comparable comparative in initial experiments, parasite burdens were determined only in the infected feet of the immunodepleted mice. For depletion of CD4 or CD8 cells, immunized mice were injected intraperitoneally with 100 µg of anti-CD4 (GK1.5) or anti-CD8 (53-5-43) antibody (eBioscience, CA) at -3 and -1 day before infection. Flow cytometry indicated that more than 95% of the target cell population was depleted. Pre-challenge immunoassays were carried out 12 weeks after the MVA boost in TRYP immunized mice.

### Cytokine, and proliferation analysis

DLN cells and splenocytes were plated at 5×10^6^/ml in RPMI (10% fetal bovine serum, 2 mM L-glutamine, 100 units/ml penicillin, 100 µg/ml streptomycin, and 50 µM β-mercaptoethanol) in 96 or 24 well plates. The cells were then stimulated with recomfbinant TRYP (5 µg/ml), recombinant LACK/p36 (5 µg/ml), soluble leishmania antigen (SLA; equivalent to 5×10^6^ parasites/ml) or left unstimulated for 72 hours. Supernatants were collected and analyzed for IFNγ, IL-10, and IL-13 using paired antibodies from BD Biosciences (CA) and R&D systems (MN). For flow cytometry, brefeldin A (BD Biosciences, CA) at 1 ug/ml was added to stimulated splenocytes during the last 4 hours, cells were surface stained with T cell markers, fixed with 2% paraformaldehyde and permeabilized with 0.05% saponin followed by intracellular staining. Isotype control antibodies were IgG1-PE-Cy7 and IgG1-PE. Forward and side scatter were used to determine lymphocytes followed by gating on CD4+ or CD8+ cells. Integrated mean fluorescence intensity (iMFI) was calculated by the following formula: iMFI  =  MFI x frequency [28]. Data were acquired using an LSRll (BD Biosciences, CA) and analyzed using FlowJo (Treestar Inc., Oregon).

For proliferation analyses to evaluate memory responses, splenic lymphocytes were labeled with 5 µM carboxyfluorescein succinimidyl ester (CFSE) at 3 months/12 weeks after the final MVA boost; cells were then placed in 96 well plates at 5×10^6^/ml in RPMI (10% fetal bovine serum, L-glutamine, penicillin/streptomycin, and β-mercaptoethanol), and stimulated with recombinant TRYP (5 µg/ml) for 3 days. Following surface staining with CD4 and CD8 antibodies, FACS analysis was done as described above to measure proliferation by dilution of CFSE dye. Unlabeled cells and unstimulated CFSE-labeled cells were used as controls.

### Antibodies and reagents

Antibodies (CD4-Pacific blue, CD8-APC, IFNγ-PE-Cy7, IL-13-PE, IgG1-PE-Cy7, IgG1-PE, affinity purified CD4 (L3T4), and CD8 (Ly-2)) were purchased from BD Biosciences (CA) and eBiosciences (CA). Pam3CSK4, CpG (ODN1826), ultra pure *E. coli* lipopolysaccharide (LPS), and MALP-2 were purchased from Invivogen Inc (CA). α-galactosyl-ceramide was obtained from Biomol International (PA).

### Ethics Statement

All experiments were approved Yale University Committee on Use and Care of Animals (Assurance number A3230-01).

### Statistics

Student's *t* test was used to determine p values indicating statistical differences for all experiments and p<0.05 was considered statistically significant.

## Results

### Adjuvant modulation of the immune response to antigen induced by DNA vaccination

Initially, we sought to examine the effects of adjuvants on the immune response induced during DNA vaccination, as it is known that priming is critical [29], , to the overall response induced in heterologous prime-boost vaccination. Compounds known to activate NK-T cells (alpha-galactosyl ceramide(α-Galcer)) [33], as well as TLR ligands TLR9 (CpG [34]), TLR2/6 (MALP-2 [35]), TLR4 (LPS [36]), and TLR2/1(Pam3CSK4 [37]) were chosen as potential adjuvants for DNA vaccination, using the *Leishmania* homologue of receptors for activated C-kinases (LACK)/p36 antigen. The adjuvants (Figure S1A) were evaluated on the basis of their ability to enhance the production of antigen specific IFNγ relative to IL-10 in DNA immunized mice, as better protection against cutaneous leishmaniasis has been associated with a higher ratio of IFNγ to IL-10 [24] and disease exacerbation of *L. (Viannia)* has been related to IL-10 levels [38]. High IL-10 production was observed for MALP-2 and LPS. The IFNγ/IL-10 ranking was Pam3CSK4≥ α-GalCer>CpG>>LPS>>>MALP-2. Consequently, amongst the adjuvants, Pam3CSK4 and α-GalCer appeared to induce a potentially useful immune response.

Based on these results, Pam3CSK4 and α-GalCer were selected to determine if a combination of Pam3CSK4 and/or α-GalCer, which have distinct cellular targets, could act synergistically to further enhance the immunogenicity of DNA-p36. In these experiments (Figures S1B and S1C), IFNγ, IL-13, and IL-10 responses to p36 were determined. Although a considerable increase in the IFNγ to IL-10 ratio was seen in response to immunization with DNA-p36+ α-GalCer +Pam3CSK4, α-GalCer alone induced increased levels of IL-13 (Figure S1C). As IL-13 has been shown to play a critical role in determining progression of non-healing pathogenesis of *L. (V.) panamensis* [22], Pam3CSK4 was selected as a potential adjuvant for use in a prime (DNA) boost (vaccinia) immunization against murine *L. (V.) panamensis* infection.

**Figure 1:**
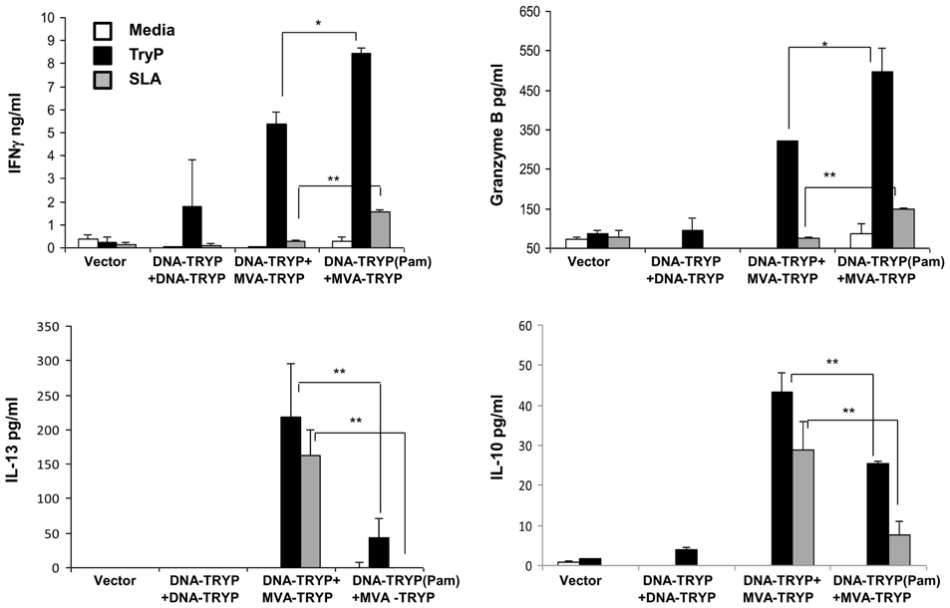
Up regulation of Th1 cytokines and down regulation of Th2 cytokines in DNA-TRYP(Pam)+MVA-TRYP immunization. Twelve weeks after the final immunization, cells from individual spleens of immunized (DNA-TRYP+DNA-TRYP, DNA-TRYP+MVA-TRYP, DNA-TRYP(Pam)+MVA-TRYP) and control mice (DNA-pVAX(Pam)+MVA-Luc) were re-stimulated *in vitro* with recombinant TRYP or SLA for 72 hours followed by analysis of supernatants obtained for IFNγ, granzyme B, IL-13 and IL-10. Data are representative of 2 experiments; n = 3 mice/group. Mean±SE. *p<0.05, **p<.005.

To further evaluate the potential of Pam3CSK4 in DNA priming, heterologous prime-boost immunization (Figure 1) using the TRYP antigen was examined. Mice were immunized intradermally with DNA-TRYP or DNA-TRYP together with Pam3CSK4. Control mice received empty vector DNA. Two weeks later, groups of the immunized mice were boosted with vaccinia virus expressing the TRYP antigen (MVA-TRYP) or with control vaccinia virus (MVA). Twelve weeks after the final immunization, the immune responses of splenocytes from immunized and non-immunized mice were analyzed. Mice immunized with DNA-TRYP(Pam3CSK4)+MVA-TRYP produced increased levels of IFNγ and granzyme B in response to both TRYP and SLA when compared to mice immunized with DNA- TRYP+MVA-TRYP or DNA-TRYP (Figure 1). Furthermore, mice receiving Pam3CSK4 during priming also produced significantly lower levels of antigen specific IL-13 and IL-10 when compared to mice immunized with DNA-TRYP+MVA-TRYP. Thus, DNA-TRYP(Pam3CSK4)+MVA-TRYP immunized mice generated higher amounts of IFNγ as well as reduced levels of IL-13 and IL-10 in response to either TRYP or SLA antigen when compared to mice immunized with DNA-TRYP alone. Hence the activation of TLR1/2(Pam3CSK4) during DNA priming (as found for DNA vaccination alone) appears to promote the down-regulation of IL-13 and IL-10 responses and concurrent up-regulation of Th1 cytokines in heterologous prime-boost vaccination, leading to higher IFNγ to IL-13 and IFNγ to IL-10 ratios.

### Pam3CSK4 during priming leads to increased antigen specific CD8 and CD4 T cells in immunized mice

**Figure 2:**
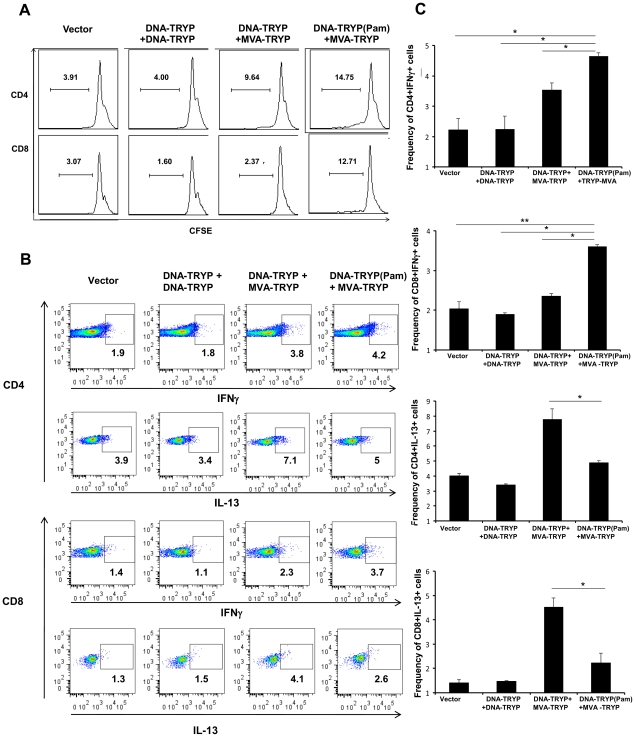
TLR2 activation leads to increased CD4+ and CD8+ memory populations. CD4 and CD8 T cell memory responses to TRYP antigen were examined 12 weeks after the MVA-TRYP boost **A**. Splenocytes from immunized and control groups of mice (3 mice/group) were CFSE labeled followed by in vitro stimulation with TRYP protein for 3 days to track proliferation of antigen specific cells. CD4 – Vector or DNA-TRYP+DNA-TRYP or DNA-TRYP+MVA-TRYP compared to DNA-TRYP(Pam)+MVA-TRYP is p<0.05. CD8 – Vector or DNA-TRYP+DNA-TRYP or DNA-TRYP+MVA-TRYP compared to DNA-TRYP(Pam)+MVA-TRYP is p<0.009. **B**. The cytokine responses of memory CD4 and CD8 cells in TRYP(Pam)+MVA-TRYP immunized mice (3 mice/group) were evaluated by FACS analysis of splenocytes stimulated *in vitro* with recombinant TRYP. Forward and side scatter were used to determine lymphocytes followed by gating on CD4+ or CD8+ cells. **C**. Overall frequency of antigen specific CD4 and CD8 cells producing IFNγ or IL-13 in different groups of vaccinated mice as determined by FACS analysis. Data are representative of two independent experiments. n = 3 mice/group.

To further evaluate the effects of Pam3CSK4 on the development of specific long-term CD4 and CD8 T cell memory, the proliferative responses to TRYP antigen were examined 12 weeks after the MVA-TRYP boost. Splenic lymphocytes from immunized mice were labeled with CFSE and then stimulated for 3 days with recombinant TRYP protein. Increased proliferation of both CD4 and CD8 cells (Figure 2A) was observed in DNA-TRYP(Pam3CSK4)+MVA-TRYP immunized mice when compared to the other vaccinated groups. Overall, the responses observed (10–15% proliferating cells) were comparable to other studies examining long-term memory [39], [40], [41]. The kinetics indicated a heightened response (CD4 and CD8 T cells) after 3 days of stimulation for the cells from mice immunized with DNA-TRYP(Pam3CSK4)+MVA-TRYP. As the number of proliferating cells is expected to be in proportion to memory populations, these results clearly indicate increased levels of both CD4 and CD8 memory cells as a consequence of vaccination using Pam3CSK4. In particular, a higher increase in antigen specific CD8 cell proliferation (5.4-fold) was observed in mice receiving DNA-TRYP(Pam3CSK4)+MVA-TRYP in comparison to CD4 cells (1.5-fold). These data suggest that although both CD4 and CD8 memory populations expand as a result of TLR1/2 ligation, a selective effect on CD8 T cell populations occurs. Overall, these data point to a more rapid and robust response and higher levels of memory populations (CD4+ and CD8+) in mice receiving Pam3CSK4 in comparison to those immunized with DNA-TRYP alone.

The immunization with DNA-TRYP(Pam3CSK4)+ MVA-TRYP resulted in a polarized Th1 immune response 12 weeks after the final booster dose (Figure 1). Given that expansion of both CD4 and CD8 memory cells (Figure 2A) was observed, it was of interest to investigate the precise cellular components of this immune response. FACS analysis of cells stimulated with recombinant TRYP was carried out at twelve weeks after the final immunization (Figure 2B). These results, consistent with ELISA results, indicated that the frequency of CD4 and CD8 cells producing IFNγ were increased in DNA-TRYP(Pam3CSK4)+MVA-TRYP immunized mice in comparison to the DNA-TRYP+MVA-TRYP immunized mice. Notably a significantly lower level of CD4 and CD8 T cells producing IL-13 was found. Overall, the increased frequency of IFNγ producing CD4 and CD8 cells in DNA-TRYP(Pam3CSK4)+MVA-TRYP immunized mice in comparison to the DNA-TRYP+MVA-TRYP immunized mice or control groups were statistically different (Figure 2C).

Overall, these results suggest that TLR1/2 activation drives the development of Th1/TC1-like responding T (CD4 and CD8) cells. Consequently, the ligation of TLR9 (by bacterial CpG sequences) together with TLR1/2 during priming appears to preferentially enhance the generation of TRYP-specific memory T cells, producing IFNγ but also significantly less IL-13.

### Protection against *L.(V).panamensis* infection requires TLR1/2 activation during priming

Given the fact that mice immunized with DNA-TRYP(Pam3CSK4)+MVA-TRYP exhibit enhanced levels of memory T cells together with an overall reduction in IL-13 and increased IFNγ production in comparison to DNA-TRYP+MVA-TRYP or DNA-TRYP vaccinated mice, we asked whether these responses might be useful in directing protection against infection. Mice were vaccinated as indicated above. Control mice were immunized with control plasmid and control MVA. Six weeks after the final immunization, all mice were infected with 5×10^4^ *L. (V). panamensis* promastigotes and lesion development was monitored.

**Figure 3:**
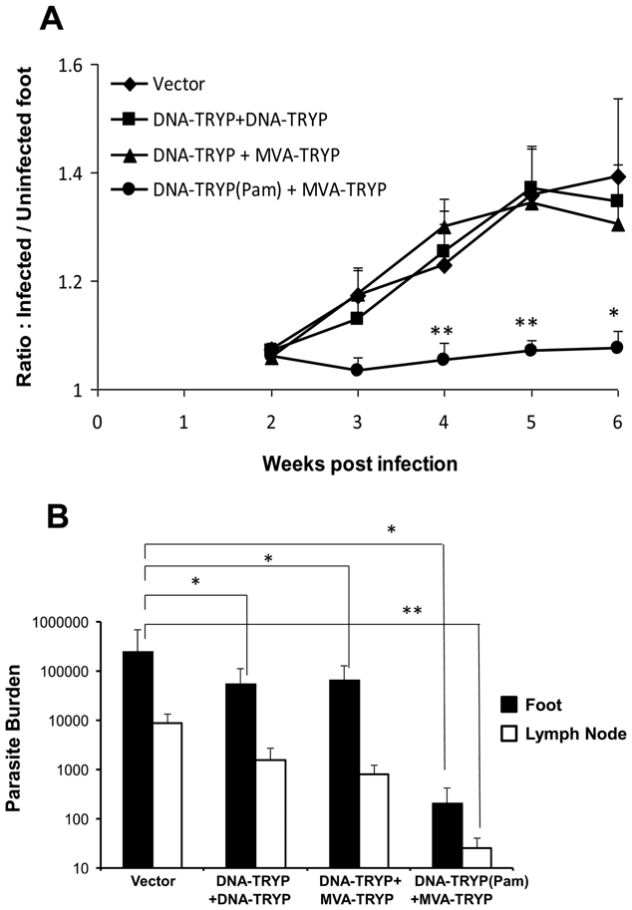
TRYP(Pam3CSK4)-MVA-TRYP protects against *L(V.) panamensis* infection. **A**. Lesion development in the control and groups of TRYP-immunized mice infected with 5×10^4^ *L (V.) panamensis*. n = 8/group For statistical significance comparison was made between DNA-TRYP(PAM3CSK4)+MVA-TRYP immunized mice and other groups of mice. **B**. Parasite burden at the site of infection and draining lymph node were determined by limiting dilution analysis at 6 weeks after infection. n = 4/group, * = p<0.05, ** = p<0.008. Results are representative of 2 independent experiments.

As shown in Figure 3A, mice immunized with DNA-TRYP(Pam3CSK4)+MVA-TRYP exhibited significantly smaller lesions when compared to control mice (control plasmid and MVA) and all other vaccine groups. Furthermore, significantly lower parasite burden levels were found at the both the site of infection and DLN of the DNA-TRYP(Pam3CSK4)+MVA-TRYP vaccinated mice, when compared to control immunized mice (574-fold) or mice immunized with DNA-TRYP (267-fold) or TRYP+MVA-TRYP (314-fold) (Figure 3B). Consistent with previously reported results [19], DNA-TRYP immunization alone failed to protect against *L.(V). panamensis* infection. Notably, heterologous prime-boost vaccination alone also does not induce protection as seen from lesion size measurement and parasite load at the site of infection. However, parasite numbers in DLN in DNA-TRYP+MVA-TRYP immunized mice are significantly lower (11-fold) than that of control mice. Therefore Pam3CSK4 plays a critical role in achieving protection against murine *L.(V). panamensis* infection using heterologous prime-boost vaccination.

### Negative role of IL-13 or IL-10 in protection against *L. (V.) panamensis* infection in mice immunized with DNA-TRYP(Pam3CSK4)+MVA-TRYP

To evaluate mechanisms underlying the protection selectively induced by DNA-TRYP(Pam3CSK4)+MVA-TRYP immunization, the immune responses in DLN cells from the *L.(V). panamensis* infected mice (control and immunized) were examined. In the control group of mice, as found for chronic infection with *L. (V.) panamensis* a mixed cytokine response was observed (IFN, IL-13, IL-10). This demonstrated an ongoing inflammatory-anti-inflammatory immune response concomitant with parasite persistence.

In general, all TRYP vaccinated groups of mice produced lower levels of cytokines than the control group of mice (vector). Although a reduction in IFNγ, as well as in IL-13 and IL-10 was observed in the DLNs of *L.(V).panamensis* infected mice immunized with DNA-TRYP(with or without Pam3CSK4)+MVA-TRYP in comparison to control mice (Table 1), the predominant effect was on the levels of IL-13 and IL-10. Interestingly the levels of IFNγ observed for vaccine groups boosted with MVA-TRYP (DNA-TRYP and DNA-TRYP(Pam3CSK4)) were comparable. However, reductions in both IL-13 and IL-10 occurred for the mice vaccinated with DNA-TRYP(±Pam3CSK4)+MVA-TRYP in comparison to those receiving DNA-TRYP alone, with the principal decrease being in IL-10 for the mice immunized with DNA-TRYP(Pam3CSK4)+MVA-TRYP.

**Table 1:** Cytokine production of vaccinated mice 6 weeks post-infection with *L.(V.) panamensis*.

Groups	IFNγpg/ml	IL-13 pg/ml	1L-10 pg/ml	Ratio IFNγ/IL-13	Ratio IFNγ/IL-10
Vector Control	7312±42	718±5.1	1057±6.1	10.2	6.9
DNA-TryP + DNA-TryP	5262±30	376±2.7	656±3.8	14.0	8.0
DNA-TryP + MVA-TryP	4797±28	177±1.3*	385±2.2*	27.2	12.4
DNA-TryP(Pam3CSK4) + MVA-TryP	4603±27	152±1.1* +	160±0.9* +	30.2	28.9

Pooled DLN cells from immunized and control mice (n = 4 mice) infected with *L.(V.) panamensis* were stimulated *in vitro* with SLA for 72 hours (duplicatè) and supernatants were analyzed for indicated cytokines by ELISA. Mean±SD. Results are representative of 2 independent experiments.

*p<0.05 DNA-TryP+DNA-TryP vs DNA-TryP+MVA-TryP or DNA-TryP(Pam)+MVA-TryP;

+ p<0.05 DNA-TryP+ MVA-TryP vs DNA-TryP(Pam)+MVA-TryP.

Although the infected vaccinated mice produced lower levels of cytokines than the control mice, it is notable that the relative levels of the cytokines differ between the various groups, with the highest IFNγ/IL-10 or IFNγ/IL-13 ratios observed for the DNA-TRYP(Pam3CSK4) +MVA-TRYP group of mice. The down-regulation of IL-10 as well as IL-13 is consistent with the memory responses observed prior to infectious challenge (Figures 1 and 2) and suggests that lower levels of these cytokines are critical to parasite containment. These results are consistent with the roles of these cytokines in pathogenesis [22]. Further, these findings are similar to vaccine studies of *L. major* utilizing MVA vaccination [24] where IFNγ/IL-10 was found to be predictive of protection.

### CD8^+^ T cells are critical to the protection induced by TLR1/2 (TRYP (Pam3CSK4)) modulation of DNA priming

**Figure 4:**
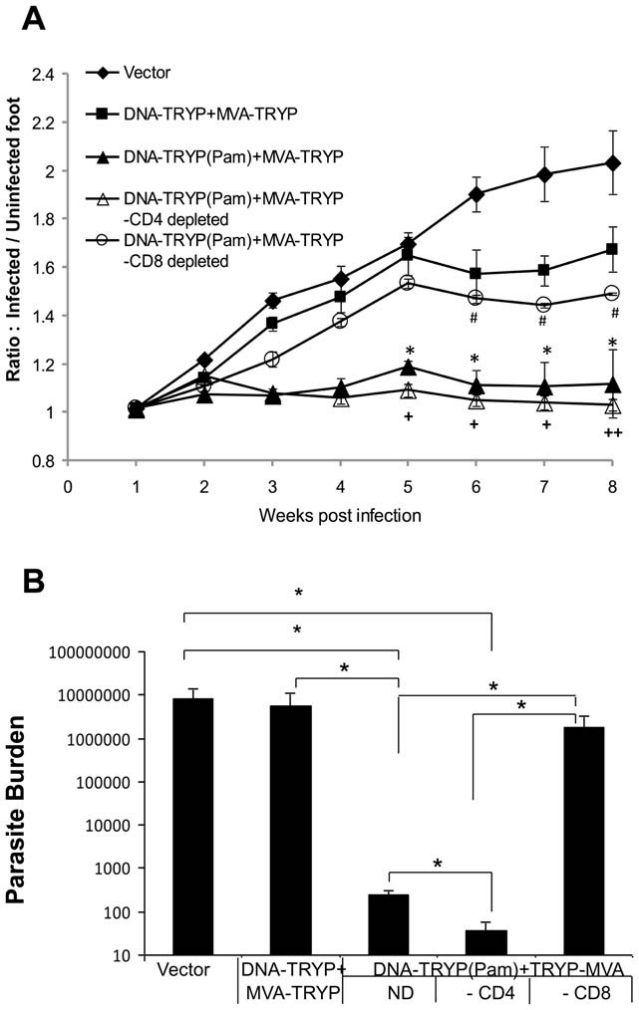
CD8 cells contribute to TLR1/2 mediated protection. **A**. Lesion size (mean±SE) progression in *L.(V).panamensis* infected mice that were protectively immunized with DNA-TRYP(Pam)+MVA-TRYP and depleted of CD4 (-CD4) or CD8 cells (-CD8) prior to infection. ND, indicates not depleted or intact vaccinated mice. Controls include mice immunized vector plasmid and MVA. n = 10 mice per group. * p<0.05 Vector vs. DNA-TRYP(Pam)+MVA-TRYP; + p<0.05 DNA-TRYP(Pam)+MVA-TRYP CD4 depleted vs. DNA-TRYP(Pam)+MVA-TRYP; ++ p<0.005 DNA-TRYP(Pam)+MVA-TRYP CD4 depleted vs. DNA-TRYP(Pam)+MVA-TRYP; # p<0.005 Vector vs. DNA-TRYP(Pam)+MVA-TRYP CD8 depleted. **B**. Parasite burden (mean± SE) at the site of infection was determined by limiting dilution assay at 8 weeks after infection. (n = 4 mice per group). * p<0.05.

The cytokine responses clearly changed with the mode of vaccination; however, it was unclear what the role of specific T cell populations might be in this process. The IFNγ responses and memory populations of both CD4 and CD8 T cells appeared to increase with TLR1/2 activation (Figure 2). To examine the specific contribution of effector CD8 and CD4 T cells to protection, vaccinated mice (DNA-TRYP(Pam3CSK4)+MVA-TRYP) were immunodepleted immediately prior to infection with *L.(V.) panamensis*. As seen in Figure 4A, CD8 T cell depletion significantly reversed protection and lesion development. Intriguingly, although lesion development was still somewhat restrained in the CD8 T cell depleted group in comparison to the vector control group, the parasite burdens in these two groups were comparable (Figure 4B), indicating no control on parasite growth occurred in the absence of CD8 T cells. The importance of CD8 T cells to protection is consistent with the heightened in vitro memory CD8 T cell expansion in response to antigen observed (Figure 2) of the mice primed with DNA-TRYP(Pam3CSK4) in comparison to mice receiving DNA-TRYP alone.

Interestingly based upon lesion development, the DNA-TRYP(Pam3CSK4)+MVA-TRYP immunized mice depleted of CD4 T cells are significantly resistant to infection. These data are confirmed by parasite burden levels at the site of infection (Figure 4B), which indicate significantly lower levels of parasites in the vaccinated mice deleted of CD4 T cells when compared to DNA-TRYP(Pam3CSK4)+MVA-TRYP immunized mice. Overall, it appears that CD8 T cell effectors in the absence of CD4 T cells can provide significant protection against *L. (V.) panamensis* infection. Furthermore, CD4 T cell effector populations do not appear to significantly contribute to protection in the vaccinated mice.

**Figure 5:**
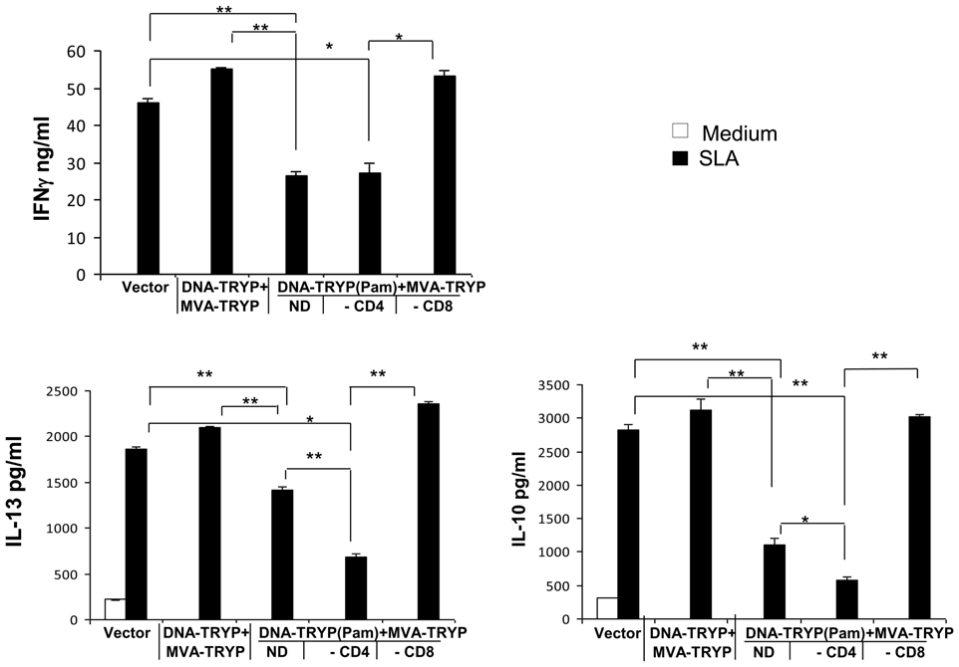
Modulation of cytokine production in TRYP(Pam)+MVA-TRYP protectively vaccinated mice depleted of CD8 or CD8 T cells. At 8 weeks post-infection with *L.(V).panamensis,* the IFNγ, IL-13 or IL-10 responses were determined in DLNs of non-immunized and TRYP immunized mice (n = 4 per group). Cells from individual mice were pooled and then stimulated for 72 hours with SLA (in duplicate) and cytokine levels determined as indicated in the Materials and Methods Section. Mean±SD. *p<0.05, ** p<0.005; -CD4, CD4 depleted; -CD8, CD8 depleted; ND – Not Depleted.

Analysis of cytokine production by DLN cells of the various groups of infected vaccinated mice indicated that the groups of protected mice (DNA-TRYP(Pam3CSK4)+MVA-TRYP and DNA-TRYP(Pam3CSK4)+MVA-TRYP–CD4 depleted) produced significantly lower levels of IFNγ, IL-13 and IL-10 (Figure 5), when compared to infected control mice. Mice immunized with DNA-TRYP+MVA-TRYP and notably mice immunized with DNA-TRYP(Pam3CSK4)+MVA-TRYP and depleted of CD8 cells had elevated levels of all three cytokines. Interestingly DNA-TRYP(Pam3CSK4)+MVA-TRYP immunized mice depleted of CD4 cells produced lower levels of IL-13 and IL-10 when compared to DNA-TRYP(Pam3CSK4)+MVA-TRYP immunized mice. However, no difference in IFNγ levels was observed between these two groups. Reduced levels of IL-10 and IL-13 have been found to lead to control of *L. (V.) panamensis* infection [22].

Consequently, it appears that healing and resolution of infection is dependent upon effector CD8 T cells; overall activation of CD8 T cells results in lower levels of IL-13 and IL-10 as well as IFNγ produced in response to infection. Interestingly, the depletion of CD4 T cells leads to a further reduction of both IL-10 and IL-13, whereas no change in the IFNγ response occurs. Together, these data demonstrate the essential role of CD8 cells in mediating protection induced by DNA-TRYP(Pam3CSK4)+MVA-TRYP against *L.(V.) panamensis* infection. Moreover, these results suggest that CD8 T cells may perform this function through the regulation of IL-13 and IL-10 production. However, further experiments are required to determine this point.

## Discussion

*The Leishmania (Viannia)* subgenus is phylogenetically divergent from other *Leishmania* [8], [9], [10]. Reflected in this is the fact that infection by members of this subgenus generates a hyperinflammatory and mixed Th1/Th2 response (and high levels of IFNγ) that can fail to resolve [11], [12], [13], [14], [15]. Furthermore vaccination against infection in the murine model has been challenging, as approaches utilizing conserved antigens previously shown to induce substantial protection against other *Leishmania sp*. have failed to provide protection [19], [21] implying that novel immunization strategies are required. Recent studies [20] indicate that immunization with replication defective *Leishmania* can provide partial protection against *L. (V.) braziliensis* infection; but the protective mechanisms involved were not explored. Our results in part are consistent with these studies, as neither DNA vaccination alone nor heterologous prime-boost vaccination (DNA+MVA) (using the conserved TRYP antigen) leads to significant protection against *L. (V.) panamensis* infection/disease. Nonetheless, the prime-boost modality has been shown to provide protection against cutaneous leishmaniasis and even the more recalcitrant visceral leishmaniasis, when DNA vaccination alone failed [42], [43]. In this report we demonstrate that significant protection against chronic *L. (V). panamensis* infection can be achieved using a heterologous prime-boost (DNA -MVA) modality using aTLR2/1 ligand (Pam3CSK4) as the adjuvant and a single defined antigen, TRYP.

As expected in response to heterologous prime-boost vaccination, groups of mice boosted with MVA-TRYP produced high levels of IFNγ. However mice immunized with DNA-TRYP(Pam3CSK4) +MVA-TRYP produced higher levels of IFNγ as well as significantly reduced levels of IL-13 and IL-10 when compared to mice immunized with DNA-TRYP+MVA-TRYP. This effect of Pam3CSK4 is consistent with initial experiments using the Pam3CSK4 adjuvant for DNA vaccination alone, thereby indicating the striking capacity of Pam3CSK4 during priming to down-regulate IL-13 and IL-10 responses, leading to a Th1 biased immune response. Interestingly Pam3CSK4 has been shown to induce a mixed cytokine response (IL-12, IL-10, TNFα) in mouse bone marrow derived dendritic cells [44]. However evidence indicates that during allergic inflammation, that enhanced Th1 responses are observed [45] in response to Pam3CSK4 stimulation. Consequently, the tissue site and context (presence of other immunomodulators) can impact the effect of an adjuvant on the developing immune response. Within the dermal compartment (site of DNA vaccination) skin resident DCs [46], [47] are the probable target population of intradermally delivered DNA-TRYP and Pam3CSK4 [48] and TLR9 (bacterial DNA-CpG)/TLR2 activation. Costimulation (TLR, NOD or TCR of NK-T cells [32], [49], [50]) of dendritic, macrophage, and early responding cells may further cooperate and selectively drive/amplify specific responses [44], [51]. DNA immunization and activation of TLR9 results in strong IL-12 production, leading to a predominant Th1 immune response [52]. Interestingly, both synergy and co-operation between TLR2 and TLR9 have been observed in response to infection [51], [53], [54] resulting in heightened Th1-like responses. Joint TLR9 and TLR2 engagement has also been shown to result in the production of MCP-1 and synergistic production of RANTES [55], which would lead to increased recruitment of macrophages/monocytes, T cells, and dendritic cells [56]. This increased cellular recruitment could potentially lead to enhanced T cell expansion, as observed herein for both CD4+ and CD8+ T cells. Further, TLR2 receptors are present on T cells and therefore TLR2 activation could also modulate the developing adaptive immune response. TLR2 activation of Tregs has been shown to mitigate (at least temporally) their suppressive quality [57], [58], [59], which could lead to increased proliferation and expansion of antigen specific T effector cells.

CD8 T cell activation has been related to both healing and pathogenesis in leishmaniasis (reviewed in [60]). In the human immune response to *Leishmania (Viannia)* infection, studies implicate CD8 T cells in disease resolution [61], [62] as well as pathology [63], [64]. The variation in the observed effects found for CD8+ T cells may reflect the functional heterogeneity of these cells [65], [66], [67]. CD8 T cells have been shown to exert a curative role in murine models of leishmaniasis, which has been attributed to the production of IFNγ [60], [68], [69], [70] as well as a potential role for perforin (CTL function)[71]. Further, other mediators produced by CD8 T cells (granzymes, chemokines, cytokines) may also contribute to the host defense. Although murine studies have unambiguously demonstrated that CD8 cells participate in vaccine-induced protection against infection [32], [68], [72] caused by other *Leishmania* species, the contributions to host defense against *Leishmania (Viannia)* have not been previously determined.

While both CD4 and CD8 T cell responses (memory and IFNγ) were increased in response to vaccination using TLR1/2, protection was largely due to the CD8 T effector cell response, as protection was reversed in mice depleted of CD8 cells, but not upon CD4 cell depletion. These results differ from heterologous prime-boost vaccination studies utilizing NK-T activation during DNA priming [32], where CD4 T responses appeared responsible for anti-leishmanial response [32]. The overall reason that CD8 T cell were the primary effectors of protection is not clear and may be due to the antigen and/or adjuvant utilized for these studies as well as the specific modes of action/effector mechanisms of the CD8 T cells elicited. It is of interest that TryP has been found in the leishmanial exosome [73], [74]. Consequently, it may be that the preferential mode of action found by CD8 T (in spite of a clear CD4 T cell response) is biased by the release and subcellular localization of this antigen during infection. However, further work is required to determine this point and the potential of exosomal antigens as CD8 vaccine candidates. Alternately, other studies employing Pam3CSK4 as an adjuvant clearly demonstrate its ability to enhance CD8 responses [75], [76], [77]. Our findings are consistent with these observations. Long-term memory (12 weeks after the vaccinia boost) development in immunized mice was heightened in mice receiving Pam3CSK4, with a marked expansion of the CD8 T cell population. Although the precise mechanism by which Pam3CSK4 augments the CD8 T cell response is not completely understood, Pam3CSK4 has been shown to enhance dendritic cell cross presentation to CD8 T cells [78]. Further, reports show that Pam3CSK4 engages TLR2 on CD8 cells [79], prolonging their survival and increasing proliferation that may contribute to increased frequency of antigen-specific CD8 cells. Furthermore, TLR2 engagement on DC subsets has been reported to lead to enhanced trafficking of these cells to the draining lymph nodes [80], [81], which would further contribute to the development of enhanced CD8 response.

We have recently shown a role for IL-13 and IL-10 in determining disease progression in the murine model of *L.(V).panamensis* [22] infection. Results presented here are consistent with this finding and further demonstrate a correlation between parasite load and levels of these cytokines. Unexpectedly, depletion of effector CD4 T cells did not appear to influence resistance. CD4 cells appear to be a source of IL-13 and IL-10 in the vaccinated mice, as observed in FACS analyses and the fact that CD4 cellular depletion results in further reduction of IL-13 and IL-10. In contrast, CD8 T effector cell depletion reversed protection and led to the production of higher levels of both IL-10 and IL-13. Overall, the consequence of CD8 cell activation was to modulate protection by limiting IL-10 and IL-13. CD8 T cells have been shown to modulate Th2 CD4 T cell function both through direct T-T interaction [82] or the indirect consequence of CD8 activation on other cell populations [83], [84]. Similarly CD8 T cell have been observed in the case of *L. major* [69] to direct increase levels of CD4-IFNγ production. Our results are consistent with the possibility that CD8 T cells are involved in the immune “deviation” of CD4 T cells and that this is involved in the development of protection against infection. However, further work is required to determine this point. An understanding of the mechanisms by which CD8 T cells promote host defense and mediate protection is of obvious interest for vaccine development against *L. (Viannia)*.

This is the first report of successful protection against *L.(V.) panamensis* using a single antigen. By utilizing a TLR2 agonist (Pam3CSK4) in a heterologous prime boost immunization method we have demonstrated protection against *L.(V.) panamensis* in a murine model. This protection was achieved specifically through the expansion of antigen-specific effector CD8 T cells. These findings suggest that the modulation of TLR1/2 signaling may dramatically improve the efficacy of DNA-based vaccine modalities, especially where CD8 T cell activation is critical, thereby contributing to effective and affordable anti parasitic vaccines.

## Supporting Information

Figure S1 **Adjuvant Modification of Cytokine Response to p36 (LACK) Antigen.** **A)** Mice (n = 3 per group) were immunized twice intradermally at biweekly intervals with either PBS (Control) or DNAp36 in combination with α-GalCer, CpG, LPS, MALP-2 or Pam3CSK4. IFN-γ and IL-10 production by splenocytes from immunized mice (stimulated in vitro with recombinant p36 three to four weeks after the last immunization) was determined. **B and C)** Mice were immunized as above using DNAp36 together with α-GalCer or/and Pam3CSK4. IFN-γ, IL-10 and IL-13 production were evaluated 3 to 4 weeks after the final immunization. Control mice received PBS alone(n = 3 per group). Results are representative of 2 experiments. Mean ± SE. The values above the bars indicate the IFN-γ/IL-10 or IFN-γ/IL-13 ratio for the specific adjuvant.
(TIF)
