# Supplementary figures and images for: TLR1/2 Activation during Heterologous Prime-Boost Vaccination (DNA-MVA) Enhances CD8+ T Cell Responses Providing Protection against Leishmania (Viannia)
Source: PLoS Negl Trop Dis. 2011 Jun 14;5(6):e1204. doi: 10.1371/journal.pntd.0001204 (PMC3114751; doi:10.1371/journal.pntd.0001204)

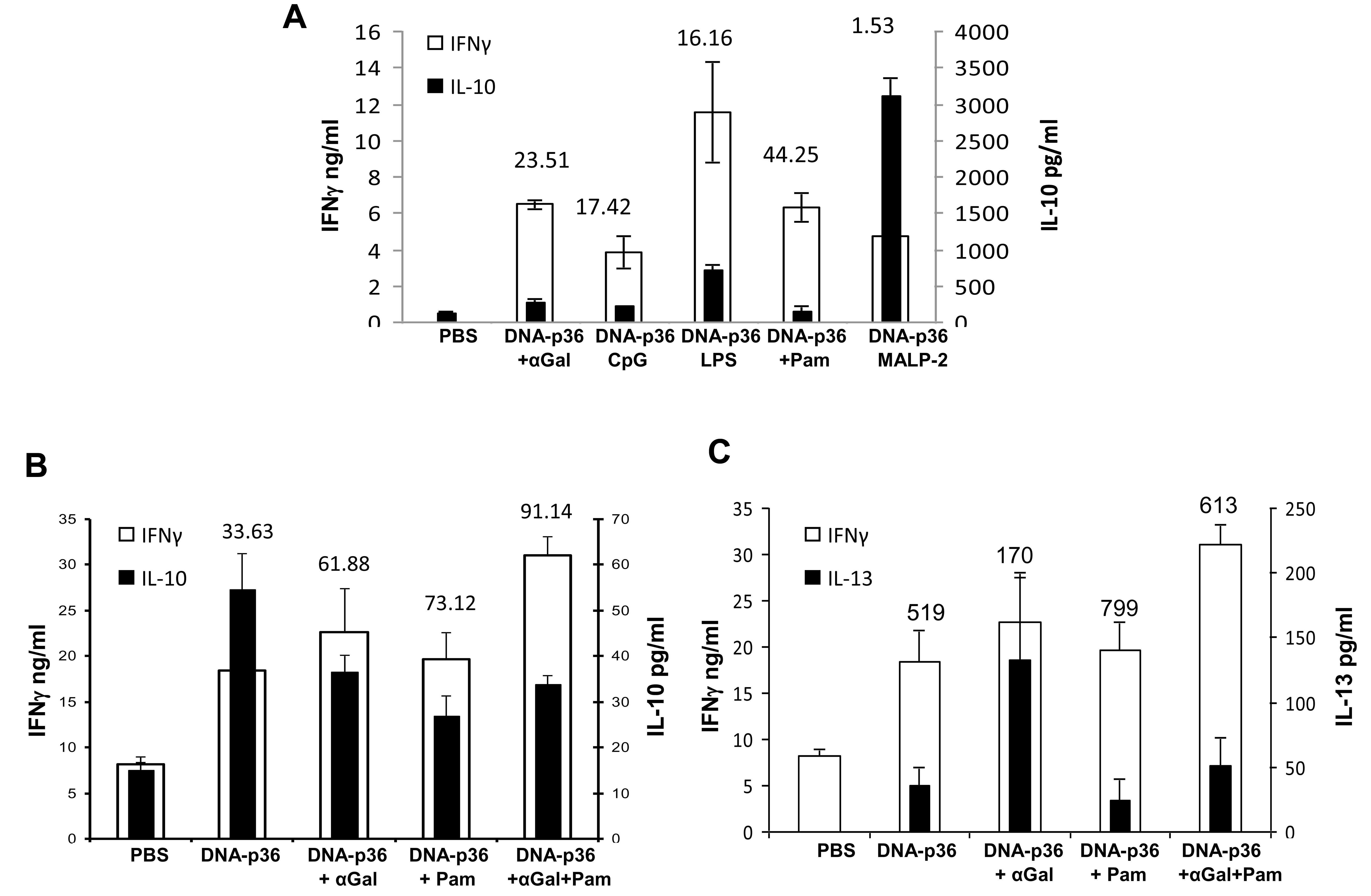

Supplement: Figure S1 — Adjuvant Modification of Cytokine Response to p36 (LACK) Antigen. A) Mice (n = 3 per group) were immunized twice intradermally at biweekly intervals with either PBS (Control) or DNAp36 in combination with α-GalCer, CpG, LPS, MALP-2 or Pam3CSK4. IFN-γ and IL-10 production by splenocytes from immunized mice (stimulated in vitro with recombinant p36 three to four weeks after the last immunization) was determined. B and C) Mice were immunized as above using DNAp36 together with α-GalCer or/and Pam3CSK4. IFN-γ, IL-10 and IL-13 production were evaluated 3 to 4 weeks after the final immunization. Control mice received PBS alone(n = 3 per group). Results are representative of 2 experiments. Mean ± SE. The values above the bars indicate the IFN-γ/IL-10 or IFN-γ/IL-13 ratio for the specific adjuvant. (TIF) [file pntd.0001204.s001.tif]
